# Supplementary material for: Structure of the [Ca]E2P intermediate of Ca2+-ATPase 1 from Listeria monocytogenes
Source: EMBO Rep. 2025 Feb 27;26(7):1709–23. doi: 10.1038/s44319-025-00392-x (PMC11977196; doi:10.1038/s44319-025-00392-x)
Supplement: Supplementary file 1 — Table EV1 [file 44319_2025_392_MOESM1_ESM.docx]

**Table EV1. RMSDs of pairwise comparison of C_α_’s of G_4_-LMCA1 [Ca]E2P to LMCA1 and SERCA structures. Underlined values indicate the closest matches to the cytosolic domain and the TM domain, respectively.** SERCA [Ca_2_]E1-AlF_x_-ADP (pdb: 1T5T) is from (Data ref: Sorensen et al., 2004); SERCA E2-BeF_x_ (pdb: 3B9B) and SERCA E2-AlF_x_ (pdb: 3B9R) are from (Data ref: Olesen et al., 2007); LMCA1 [H]E2-BeF_x_ (pdb: 6ZHF) and LMCA1 [H]E2-AlF_x_ (pdb: 6ZHG) are from (Data ref: Hansen et al., 2021).

| **Molecule 1** | **Molecule 2** | **Full molecule** | ***Cytosolic domains** | ****TM domain** |
| --- | --- | --- | --- | --- |
| G_4_-LMCA1  [Ca]E2P | SERCA [Ca_2_]E1- AlF_x_-ADP (pdb: 1T5T) | 7.6 Å | 12.2 Å | 2.1 Å |
|  | LMCA1 [H]E2-BeF_x_ (pdb: 6ZHF) | 4.2 Å | 2.9 Å | 3.3 Å |
|  | SERCA E2-BeF_x_ (pdb: 3B9B) | 3.5 Å | 1.9 Å | 5.0 Å |
|  | LMCA1 [H]E2-AlF_x_ (pdb: 6ZHG) | 4.7 Å | 3.0 Å | 3.4 Å |
|  | SERCA [H_2-3_]E2-AlF_x_ (pdb: 39BR) | 5.0 Å | 3.1 Å | 4.2 Å |

* The cytosolic domains (A, P, N) are defined as:

LMCA1: Residue 1-35 and 112-225 and 314-655
SERCA: Residue 1-37 and 124-241 and 331-735

** The TM domains (M1 through M10) are defined as:

LMCA1: Residue 36-111 and 226-313 and 656-880

SERCA: Residue 38-123 and 242-330 and 736-994
