# Supplementary material for: Structure of the [Ca]E2P intermediate of Ca2+-ATPase 1 from Listeria monocytogenes
Source: EMBO Rep. 2025 Feb 27;26(7):1709–23. doi: 10.1038/s44319-025-00392-x (PMC11977196; doi:10.1038/s44319-025-00392-x)
Supplement: Supplementary file 2 — Table EV2 [file 44319_2025_392_MOESM2_ESM.docx]

**Table EV2. Data collection and refinement for cryo-EM single particle analysis.**

| **Data collection and processing** |  |
| --- | --- |
| Magnification | 130,000x |
| Voltage (kV) | 300 |
| Microscope | Titan Krios G3i |
| Camera | Gatan K3 |
| Physical pixel size (Å/pix) | 0.647 |
| Electron exposure (e-/Å^2^) | 59.0 (grid 1+2), 58.5 (grid 3+4) |
| Defocus range (µm) | -0.8 to -2.0 |
| Total number of movies | 13,273 |
| Initial particles | 3,001,118 |
| Final particles | 101,034 |
| Symmetry imposed | C1 |
| Map resolution (Å) | 3.46 |
| FSC threshold | 0.143 |
| **Refinement** |  |
| Initial model used | Fusion between ‘pdb: 6ZHH’ and LMCA1 homology of ‘pdb: 1T5T’ |
| Atoms | 13618 (Hydrogens: 6901) |
| Residues | 880 |
| Ligands | 2 (Ca^2+^, Mg^2+^) |
| Average B factor (Å^2^) | 83.32 |
| Bond length RMSD (Å) | 0.003 |
| Bond angle RMSD (°) | 0.618 |
| MolProbity score | 2.07 |
| Clashscore | 13.37 |
| Ramachandran outliers (%) | 0 |
| Ramachandran allowed (%) | 6.76 |
| Ramachandran favored (%) | 93.24 |
| PDB code | 9GQO |
| EMDB code | EMD-51510 |
