# Supplementary material for: Structure of the [Ca]E2P intermediate of Ca2+-ATPase 1 from Listeria monocytogenes
Source: EMBO Rep. 2025 Feb 27;26(7):1709–23. doi: 10.1038/s44319-025-00392-x (PMC11977196; doi:10.1038/s44319-025-00392-x)
Supplement: Supplementary file 4 — Expanded View Figures [file 44319_2025_392_MOESM4_ESM.pdf]

Expanded View Figures

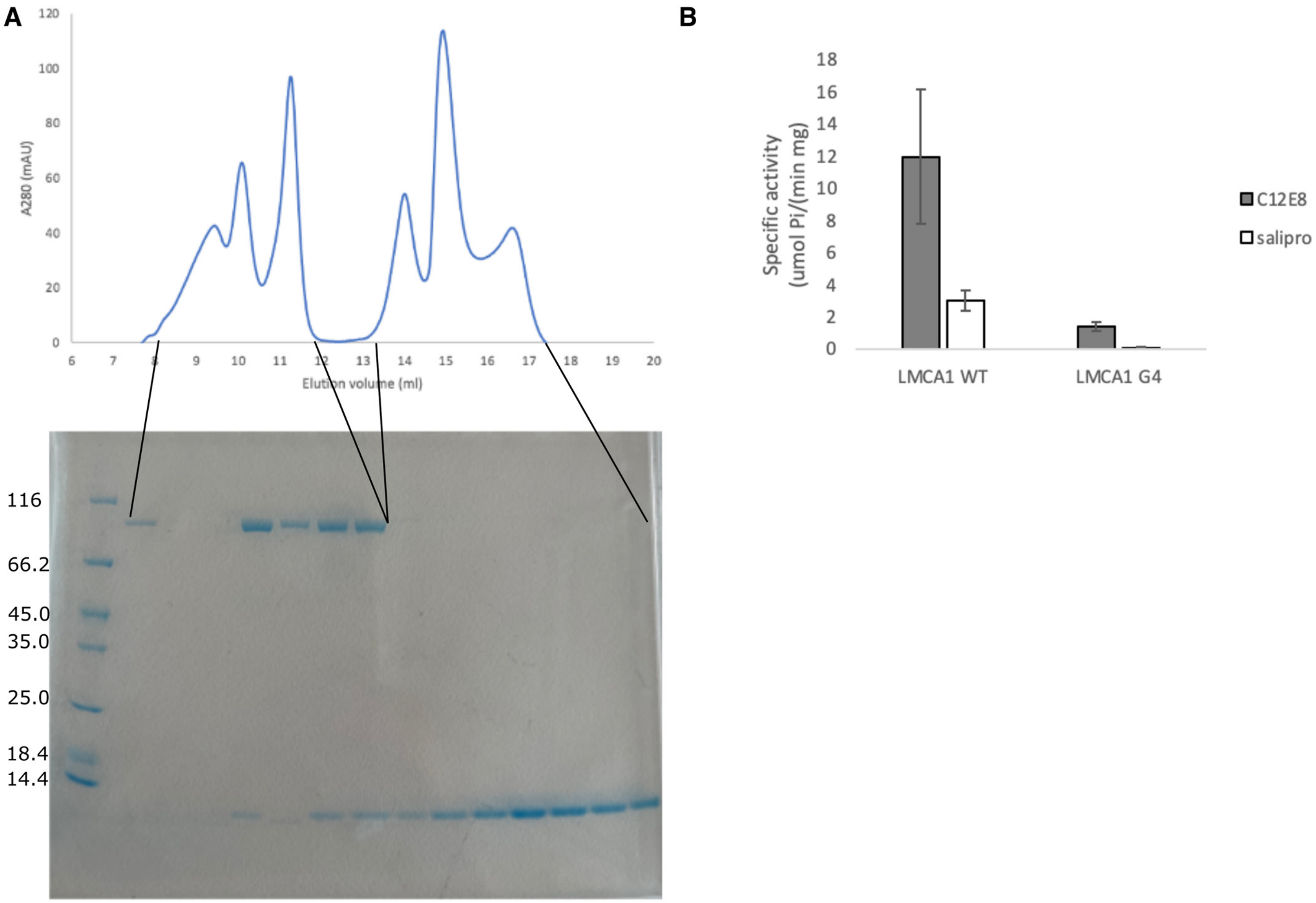

Figure EV1. Salipro reconstitution and activity.

(**A**) Chromatogram of salipro reconstitution of LMCA1. The peak eluting at 11–12 ml contains monomeric LMCA1 in nanodiscs. (**B**) Specific ATPase activity is obtained by linear regression of a time-course measurement. Error bars represent the standard deviation of three technical replicates.

 © The Author(s)

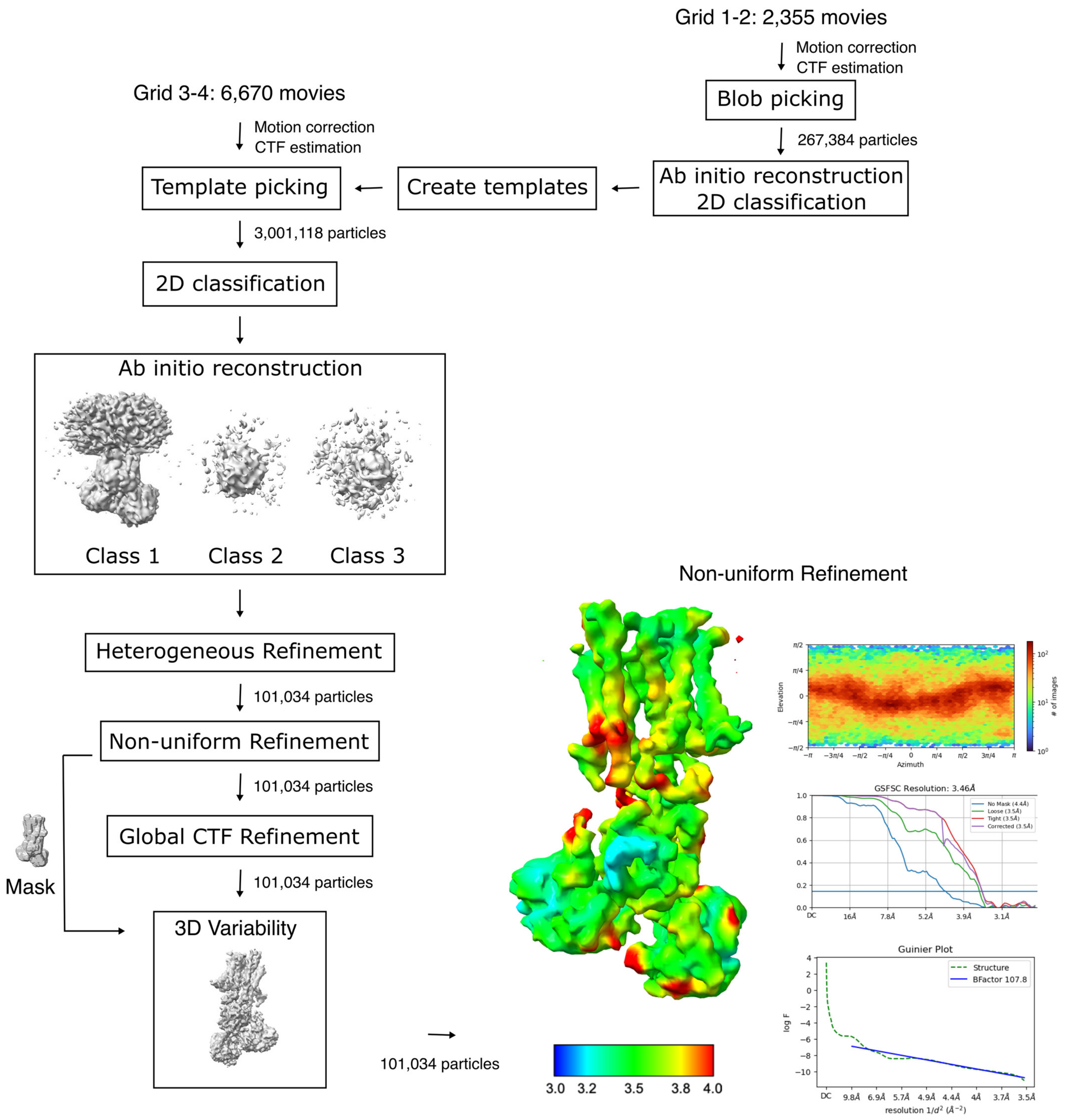

Figure EV2. The CryoSPARC data processing pipeline for cryo-EM single particle analysis.

Data from four grids were merged into a single dataset and processeed. The final struture from a non-uniform reefinement is shown with its local resolution represented by a various colors. Plots from the final refinement are shown.

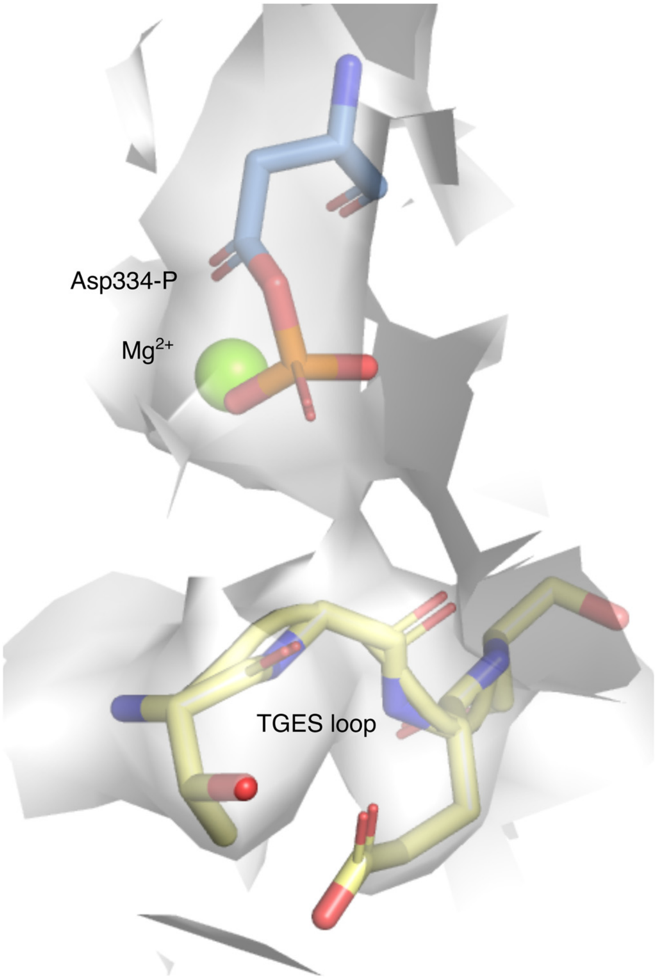

Figure EV3. Density for the phosphorylation site.

The catalytic aspartate (Asp334) is phosphorylated and coordinated by a Mg^{2+} ion. The TGES loop is protecting the phosphorylation site. Density is only shown for the TGES loop in yellow, Asp334 in blue, the phosphorylation and the Mg^{2+} ion.

 © The Author(s)

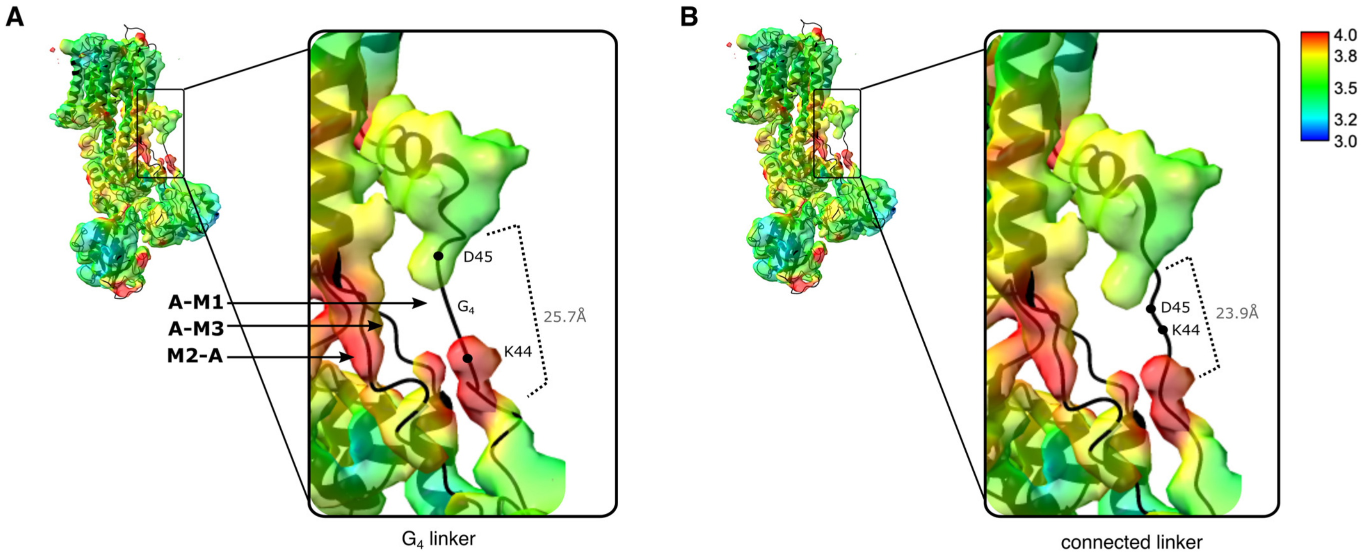

Figure EV4. The A-M1 linker can span the domains in a strained conformation in WT LMCA1 in [Ca]E2P.

(**A**) The break in linker A-M1 is indicated with numbered residues, and the C_αLeu39 - C_αPro46 distance is measured. (**B**) Lys44 and Asp45 can connect without the G_4 insert by remodeling the loop in coot. The linker can span the domains in an extended conformation if a local deviation from the density is allowed. The C_αLeu39 - C_αPro46 distance is measured like in Fig. 4. The map is shown at $\sigma = 0.225$.
